# Characterization of mouse ocular response to a 35-day spaceflight mission: Evidence of blood-retinal barrier disruption and ocular adaptations

**DOI:** 10.1038/s41598-019-44696-0

**Published:** 2019-06-03

**Authors:** Xiao W. Mao, Nina C. Nishiyama, Stephanie D. Byrum, Seta Stanbouly, Tamako Jones, Alyson Drew, Vijayalakshmi Sridharan, Marjan Boerma, Alan J. Tackett, David Zawieja, Jeffrey S. Willey, Michael Delp, Michael J. Pecaut

**Affiliations:** 1 Department of Basic Sciences, Division of Biomedical Engineering Sciences (BMES), Loma Linda University School of Medicine and Medical Center, Loma Linda, CA 92350 USA; 2 Department of Biochemistry and Molecular Biology, University of Arkansas for Medical Sciences, Little Rock, AR 72202 USA; 3 Arkansas Children’s Research Institute, Little Rock, AR USA; 4 Division of Radiation Health, Department of Pharmaceutical Sciences, University of Arkansas for Medical Sciences, Little Rock, AR 72202 USA; 5 Department of Medical Physiology, Texas A&M University, College Station, Texas USA; 6 Department of Radiation Oncology, Wake Forest School of Medicine, Bowman Gray Center, Winston-Salem, NC 27101 USA; 7 Department of Nutrition, Food and Exercise Sciences, Florida State University, Tallahassee, FL 32306 USA

**Keywords:** Proteomic analysis, Molecular neuroscience

## Abstract

The health risks associated with spaceflight-induced ocular structural and functional damage has become a recent concern for NASA. The goal of the present study was to characterize the effects of spaceflight and reentry to 1 g on the structure and integrity of the retina and blood-retinal barrier (BRB) in the eye. To investigate possible mechanisms, changes in protein expression profiles were examined in mouse ocular tissue after spaceflight. Ten week old male C57BL/6 mice were launched to the International Space Station (ISS) on Space-X 12 at the Kennedy Space Center (KSC) on August, 2017. After a 35-day mission, mice were returned to Earth alive. Within 38 +/− 4 hours of splashdown, mice were euthanized and ocular tissues were collected for analysis. Ground control (GC) and vivarium control mice were maintained on Earth in flight hardware or normal vivarium cages respectively. Repeated intraocular pressure (IOP) measurements were performed before the flight launch and re-measured before the mice were euthanized after splashdown. IOP was significantly lower in post-flight measurements compared to that of pre-flight (14.4–19.3 mmHg vs 16.3–20.3 mmHg) (p < 0.05) for the left eye. Flight group had significant apoptosis in the retina and retinal vascular endothelial cells compared to control groups (p < 0.05). Immunohistochemical analysis of the retina revealed that an increased expression of aquaporin-4 (AQP-4) in the flight mice compared to controls gave strong indication of disturbance of BRB integrity. There were also a significant increase in the expression of platelet endothelial cell adhesion molecule-1 (PECAM-1) and a decrease in the expression of the BRB-related tight junction protein, Zonula occludens-1 (ZO-1). Proteomic analysis showed that many key proteins and pathways responsible for cell death, cell cycle, immune response, mitochondrial function and metabolic stress were significantly altered in the flight mice compared to ground control animals. These data indicate a complex cellular response that may alter retina structure and BRB integrity following long-term spaceflight.

## Introduction

Astronaut health and safety are of key importance to the success of long-term missions in space. More than 50% of the astronauts returning from space shuttle missions or the International Space Station (ISS) have reported a subjective change in their visual acuity^1^. The underlying mechanisms and pathological manifestations of increased vision impairment during and after spaceflight is currently unknown. Various factors have been suggested to account for these disturbances, including increased intracranial pressure (ICP). Modeling studies have shown that a compromise in the integrity of the cerebrovascular barrier function would serve to elevate ICP^2^. Similar to how the blood-brain barrier (BBB) functions in the cranium, the blood-retinal barrier (BRB) is an endothelial barrier which regulates vascular permeability and maintains intraocular pressure (IOP). Little work has been done to examine retinal structure and BRB integrity/function following prolonged exposure to spaceflight in animal models.

Spaceflight has been reported to have numerous negative effects on ocular and structure and function^3^. However, data on how spaceflight impacts protein expression in the retina are limited. We have previously evaluated ocular tissue responses obtained from mice flown to and from the ISS on both the Space Shuttle Atlantis (STS-135)^4^ as well as the SpaceX-9 Dragon rocket^5^. Although numerous differences were noted in the flight mice compared to control animals on ground, an intriguing finding especially relevant to the present study was that spaceflight conditions induced significant changes in protein expression related to endothelial apoptosis, inflammation and metabolic function in the retina.

The goal of the present study was to characterize the effects of spaceflight on retinal vascular alterations and BRB integrity, and to identify changes in protein expression profiles in mouse ocular tissue following spaceflight using immunohistochemistry (IHC) and a proteomics approach.

## Results

### IOP measurement

**Figure 1:**
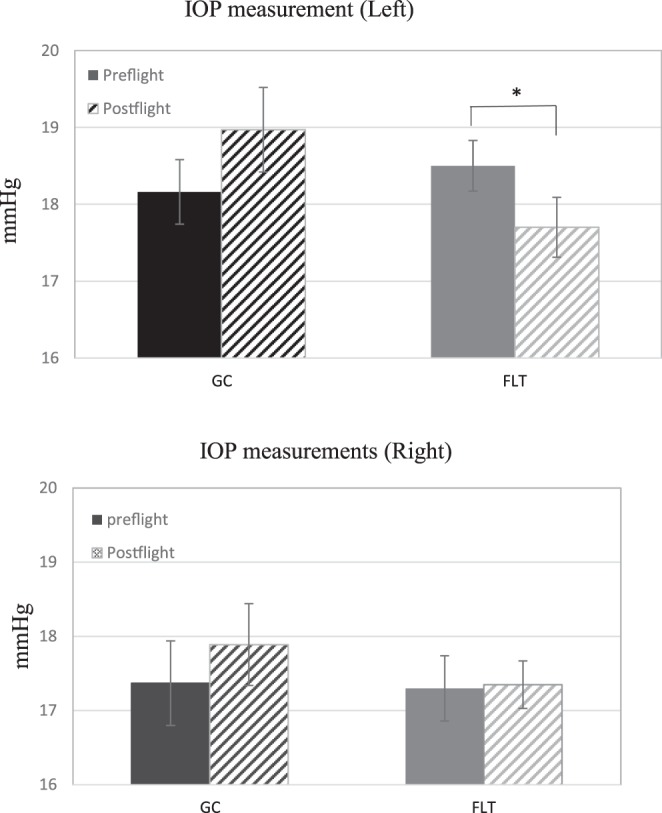
Intraocular pressure (IOP) measurements. (**A**) Mean pre-and post-flight IOP for flight (FLT) and ground control (GC) mice for left eyes (n = 20). (**B**) Mean pre-and post-flight IOP for FLT and GC mice for right eyes (n = 20). The animals exhibited no signs of irritation or discomfort during the procedure. Six readings were made to obtain an average count as a single IOP readout. Three IOP measurements were obtained for each eye. Values are means ± SEM. ^*^Significantly lower than that of preflight (p < 0.05).

Tukey’s post hoc comparison showed that IOP was significantly lower in post-flight measurements compared to that of pre-flight (14.4–19.3 mmHg vs 16.3–20.3 mmHg, p < 0.05) in the left eye of the flight (FLT) mice (Fig. 1A). However, there were no significant differences in IOP measurements in the right eye (Fig. 1B).

### Apoptotic damage in the retina

**Figure 2:**
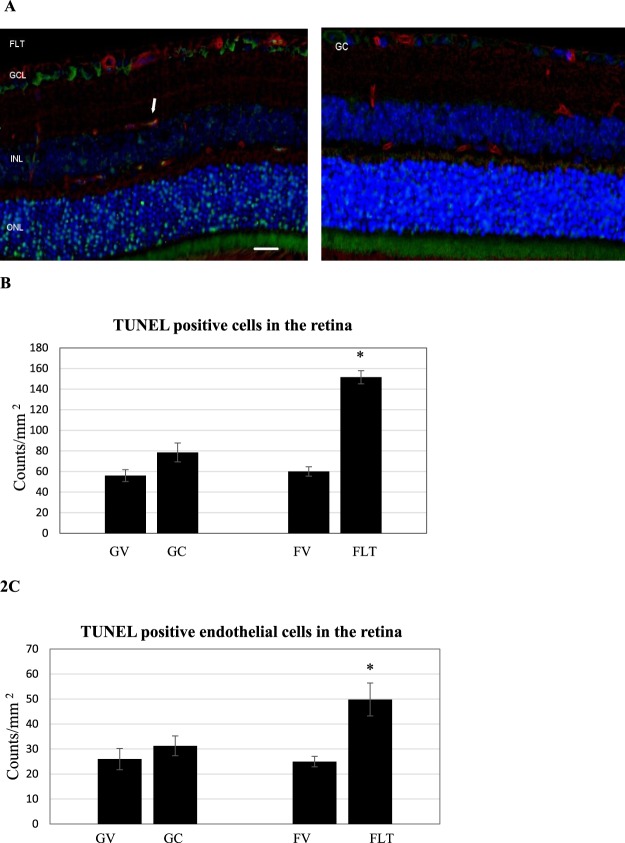
Apoptosis based on terminal deoxynucleotidyltransferase dUTP nick-end labeling (TUNEL) staining of male C57BL/6 flight (FLT), ground control (GC) mouse retinal tissue. (**A**) TUNEL-positive cells were identified with green fluorescence, endothelium was stained with lectin (red). TUNEL-positive cells that were laid within red lectin-labeled endothelium were identified as TUNEL-positive endothelial cells. The nuclei of photoreceptors were counterstained with DAPI (blue). In the control retinal tissue, only sparse TUNEL-positive cells were found. In the retina from flight mice, TUNEL-positive labeling was apparent in the retinal endothelial cells, nuclear layers and ganglion cell layers. Arrow: TUNEL-positive endothelial cell. Outer nuclear layer (ONL); inner nuclear layer (INL); ganglion cell layer (GCL). Scale bar = 50 μm. (**B**) Apoptotic cell density in the retinal ONL, INL and GCL of ground vivarium (GV), Ground control (GC), flight vivarium (FV) and Flight (FLT) mice; (**C**) Apoptotic cell density in the retinal endothelium of GV, GC, FV and FLT mice. Values are represented as mean density ± SEM for 6 mice/group, and the density profiles were expressed as mean number of apoptotic positive cells/mm^2^. The mean of the density profile measurements across 5 retina sections per eye was used as a single experimental value. Values are represented as mean density ± SEM for 6 mice/group. ^*^Significantly higher than all other groups (p < 0.05).

Representative images of terminal deoxynucleotidyl transferase dUTP nick-end labeling (TUNEL) assay were presented for FLT, habitat ground controls (GC) and vivarium control conditions, including both flight vivarium (FV) and ground vivarium (GV) control groups (Fig. 2A). Quantitative assessment indicated that the density of apoptotic cells in the retinal nuclear layer and ganglion cell layer (GCL) was significantly higher in the FLT group, and was 2–2.5 fold higher compared to that in the GC group (Fig. 2B). The number of TUNEL positive endothelial cells were also calculated (Fig. 2C). A significant increase of apoptosis in retinal endothelial cells was detected ranging from 1.6 to 2.0-fold for FLT group compared to ground and vivarium controls, respectively (p < 0.05).

### BRB integrity and endothelial proteins

**Figure 3:**
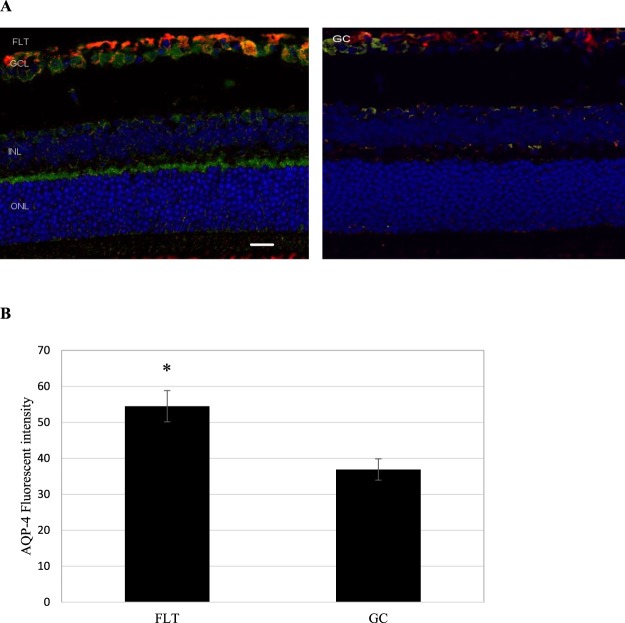
Glial fibrillary acidic protein (GFAP) and aquaporin-4 (AQP-4) staining in the retina. (**A**) Representative micrographs of ocular sections after immunostaining with anti-GFAP and AQP-4 antibodies on flight (FLT) and ground control (GC) samples. AQP-4 positive staining is identified by green fluorescence, GFAP with red, and the cell nuclei with blue (DAPI). Increased AQP-4 staining was seen in the FLT group compared with controls. No significant differences in GFAP staining was noted between two groups. Scale bar = 50 μm. (**B**) The average fluorescence intensity for AQP-4 was measured and calculated using the ImageJ program. Fluorescence was averaged across 6 retinas per group. Values are represented as mean density ± SEM. ^*^Significantly higher than ground control (GC) groups (p < 0.05).

Two astrocyte markers were used to examine the integrity of the BRB: glial fibrillary acidic protein (GFAP) and aquaporin-4 (AQP-4). GFAP is present in the cell bodies and large processes of astrocytes. It is an intermediate filament protein involved in the maintenance of the BRB. (Fig. 3A). AQP-4 is a water channel protein concentrated at the luminal surfaces of astrocyte end-feet which clearly outline the vascular bed to which they adhere. In the retina, AQP4 is expressed in glia cells (astrocyte and Müller cells) and is responible for BRB integrity. Increased positive AQP4 staining (green) was seen in the retinal GCL and inner nuclear layer (INL) of the FLT group (Fig. 3A) compared with ground controls. As shown in Fig. 3B, the fluorescent intensity reflecting endogenous levels of AQP-4 in the retina was significantly greater in the FLT group compared with the GC group (p < 0.01). There was no signficant differences between three control groups; the average intensities were 36.9 +/− 3.0, 34.9 +/− 1.9 and 35.1 +/− 3.6 for the GC, GV and FV groups respectively. There was no notable difference in the pattern and instensity of GFAP staining.

**Figure 4:**
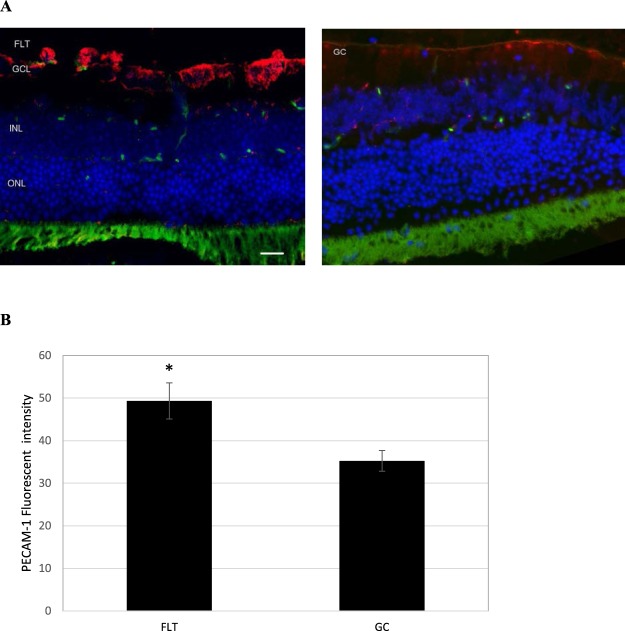
Platelet endothelial cell adhesion molecule (PECAM-1) staining in the retina. (**A**) Representative images of PECAM-1 ocular sections of flight (FLT) and ground control (GC) mice. PECAM-1 positive cells were identified with red fluorescence, endothelium was stained with lectin (green). The nuclei of photoreceptors were counterstained with DAPI (blue). In the control retinal tissue, only some positive cells were found. In the retina from flight mice, enhanced immunoreactivity of PECAM cells was apparent in the retinal inner nuclear layer (INL) and ganglion cell layers (GCL). Scale bar = 50 μm. (**B**) Immunoreactivity of PECAM-1 staining in the retina. The average fluorescence intensity for PECAM-1 activity was measured and calculated using the ImageJ program. Fluorescence was averaged across 5 retinas per group. Values are represented as mean density ± SEM. ^*^Significantly higher than GC group (p < 0.05).

**Figure 5:**
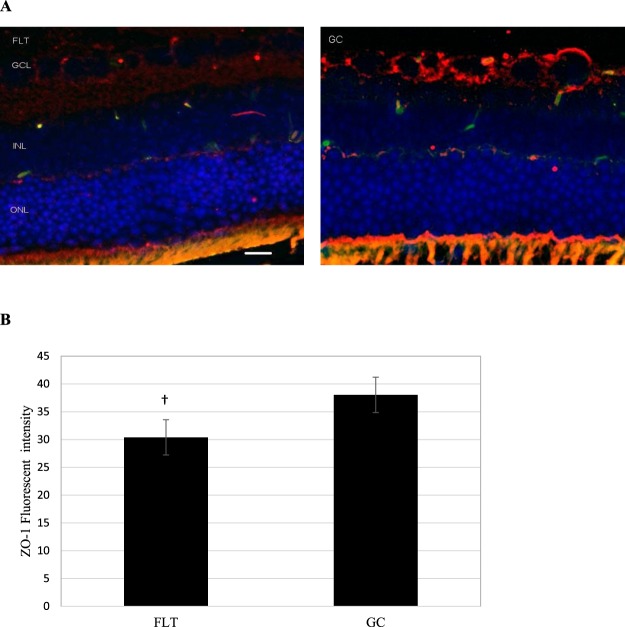
Zonula occludens-1 (ZO-1) staining in the retina. (**A**) Representative images of ZO-1 in ocular sections of flight (FLT) and ground control (GC) mice. ZO-1 positive cells were identified with red fluorescence, endothelium was stained with lectin (green). The nuclei of photoreceptors were counterstained with DAPI (blue). In the ground control (GC) retinal tissue, positive ZO-1 staining were apparent in the retinal inner nuclear layer (INL) and ganglion cell layer (GCL). Only some-positive cells were found in the retina from FLT mice. Scale bar = 50 μm. (**B**) Immunoreactivity of ZO-1 staining in the retina. The average fluorescence intensity for ZO-1 was measured and calculated using the ImageJ program. Fluorescence was averaged across five retinas per group. Values are represented as mean density ± SEM. ^†^Tendency for difference between FLT and GC groups (p = 0.062).

To examine whether spaceflight alters cellular expression of the endothelial cell juction proteins and retinal barrier integrity, the eye tissue expression of the endothelial cell-cell adhesion molecule, platelet endothelial cell adhesion molecule (PECAM-1) and tight junction (TJ) proteins, such as Zonula occludens-1 (ZO-1) were evaluated. In FLT mice, increased immunoreactivity for PECAM-1 (red) was predominantly present in GCL (Fig. 4A). PECAM-1 immunoreactivity was significantly higher in the FLT group compared to GC mice (Fig. 4B). Furthermore, ZO-1 expression (red) was detected at the GCL and the photorecepotor inner segment (IS) of the eye tissues (Fig. 5A). The level of ZO-1 immunoactivity tended to be lower (p = 0.062) in the FLT group compared to that in GC controls (Fig. 5B).

### Proteomics analysis of mouse ocular tissues

**Figure 6:**
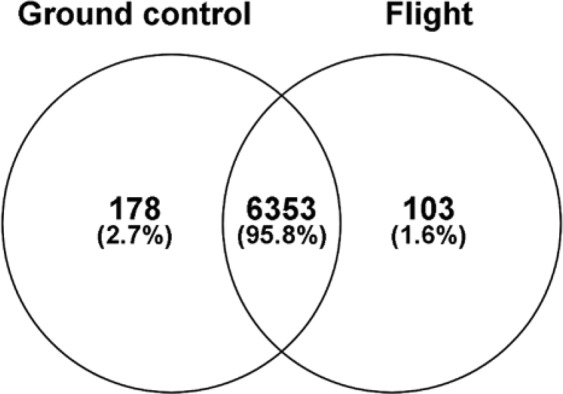
Venn diagram of the total number of proteins identified in the retinas of ground control and flight mice. 95.8% of the proteins were identified in both groups, while 2.7% and 1.6% of proteins identified were unique to ground control and flight, respectively.

Twenty micrograms of protein from retina samples in the GC and FLT groups were resolved by 4–20% sodium dodecyl sulfate (SDS) Tris-Gly gel electrophoresis, visualized by Coomassie stain, in-gel trypsin digested, and analyzed by LC/MS on an Orbitrap Fusion Tribrid mass spectrometer. A MaxQuant database search (restricted to *Mus musculus*) identified a total of 6,634 proteins from all 12 samples with 6,353 (96%) proteins identified in common among the GC and FLT samples (Fig. 6). MaxQuant iBAQ intensity values were median normalized, log_2_ transformed, and missing values were imputed based on the normal distribution using Perseus (version 1.5.6.0)^6^. Batch effects were corrected using the ComBat function from the sva bioconductor package^7^. The log_2_ normalized iBAQ intensity values were used for statistical analysis. A Mann-Whitney U test was performed and p-values were adjusted using the Benjamini-Hochberg procedure to control the FDR level.

**Figure 7:**
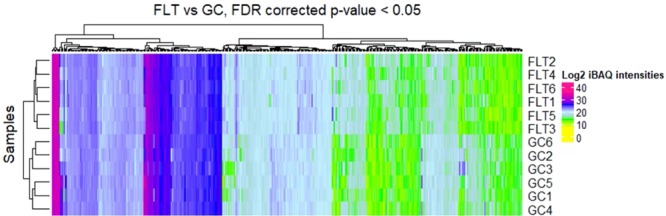
Hierarchical cluster of significantly differentiating proteins between ground control (GC) and flight (FLT). The log_2_ normalized iBAQ intensities are shown. Proteins were considered significant with a FDR adjusted p-value < 0.05 and are clustered along the top of the heatmap. The proteins clearly separate the two sample groups.

**Figure 8:**
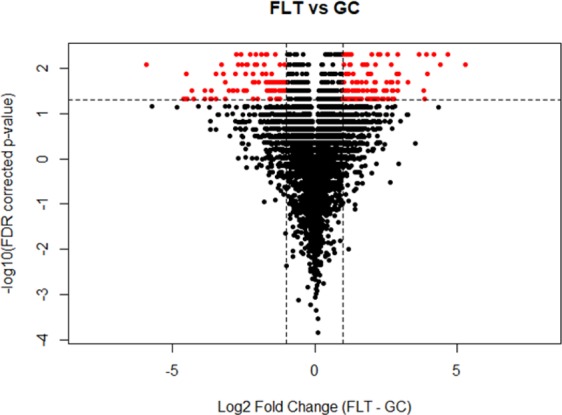
Volcano plot showing all 6,634 identified proteins in the retina of ground control and flight mice. The y-axis consists of −log_10_ *p*-values based on the Mann–Whitney U FDR adjusted *p*-values, while the *x*-axis consists of the log_2_ fold change. The vertical lines indicate a fold change >2 threshold. The horizontal line indicates a *p*-value of 0.05. Proteins highlighted in red were significant at a fold change >2 and an adjusted p-value < 0.05. Proteins in the upper left quadrant have an increased expression in the ground control (GC) group, whereas proteins in the upper right quadrant are increased in flight (FLT) group.

**Figure 9:**
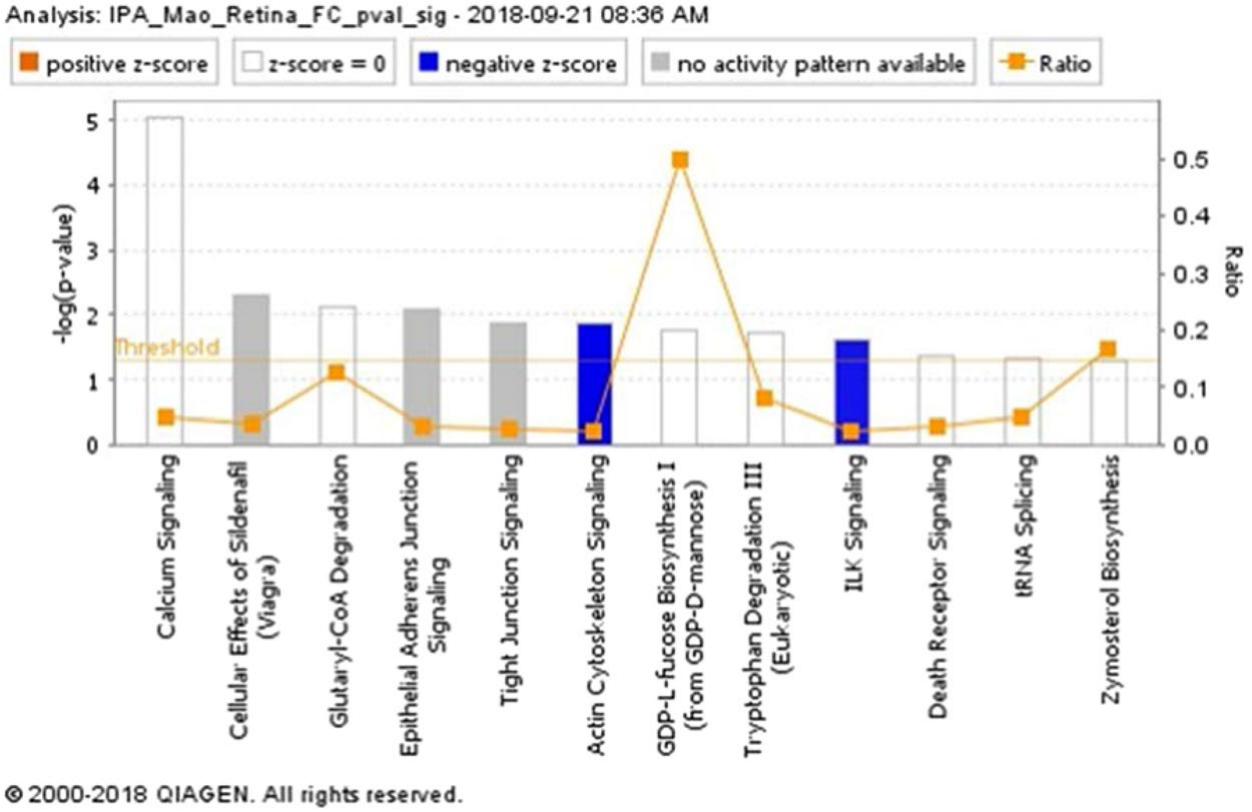
Canonical pathways from Ingenuity Pathway Analysis (IPA, https://www.qiagenbioinformatics.com/products/ingenuitypathway-analysis) of the differentially expressed proteins between ground control and flight. Pathways were considered significant based on the Fisher exact test with a −log_10_ *p*-value > 1.3 (corresponds to a *p*-value < 0.05). The number of proteins that map to each pathway followed by the number of total proteins identified in each pathway were listed as follows: Calcium Signaling = 10/206; Cellular Effects of Sildenafrl = 5/131; Glutaryl-CoA Degradation = 2/16; Epithelial Adherens Junction Signaling = 5/150; Tight Junction Signaling = 5/167; Actin Cytoskeleton Signaling = 6/233; GDP-L-fucose biosynthesis I = 1/2; Tryptophan Degradation III = 2/25; ILK signaling = 5/197; Death receptor signaling = 3/93; tRNA splicing = 2/42; Zymosterol Biosynthesis = 1/6.

We identified a total of 6,634 proteins of which 485 significantly differentiated in FLT compared to GC by FDR adjusted p-value < 0.05 (Supplementary Table 1). These proteins are shown as a heatmap using a hierarchical cluster of the log_2_ normalized iBAQ intensities for all samples (Fig. 7). The significant protein profile is able to clearly separate the two groups. This list was then reduced to 224 significant proteins when we included a fold change >2 cutoff (Supplementary Table 2). Volcano plots were generated to visualize all identified proteins and highlight significant proteins. The y-axis consists of −log_10_ *p*-values based on the Mann–Whitney U FDR adjusted *p*-values, while the *x*-axis consists of the log_2_ fold change. The vertical lines indicate up- and down expression using a fold change >2 threshold. The horizontal line indicates a *p*-value of 0.05 (Fig. 8). A total of 126 proteins were up-regulated while 98 proteins were down-regulated in FLT compared to GC. Ten most significantly increased and decreased protein expression levels related to cellular organization, cell cycle, apoptosis, circadian clock, neuronal and mitochondrial function are summarized and listed in Table 1. These differentially expressed proteins were then analyzed using the Ingenuity Pathway Analysis (IPA, https://www.qiagenbioinformatics.com/products/ingenuitypathway-analysis)^8^. Pathways were considered significant based on the Fisher exact test with a −log10 p-value > 1.3 (corresponds to a p-value < 0.05). IPA revealed significant changes in pathways related to cell death, adhesion juction/tight junction signaling, calcium signaling, mitochondrial function and metabolism. Significantly affected canonical pathways between GC and FLT groups are shown in Fig. 9.

**Table 1:** Most up and down differentially expressed proteins identified by IPA in the mouse retina in response to flight (FLT) vs ground controls (GC); fold change >2; FDR corrected p < 0.05.

Proteins	Fold changes	Function
FAM98B	5.59	Cell organization
PTN	5.404	Apoptotic signaling
Tpm1	5.30	Cell structure and function
MRPL42	5.195	Mitochondrial function
PMEL	5.02	Cellular organization
LYRM9	4.609	Cell assembly
S100A1	4.55	Cell cycle
TUSC2	4.43	Cell cycle
FAM210B	3.78	Cell cycle progression
SCYL3	−6.79	Neuron development and viability
CRYBB1	−5.28	Ganglion cell survival
Gm1553	−5.14	Immune response
ISY1-RAB3	−4.10	Cell organization
TNNT 3	−3.93	Calcium signaling
EPB 41	−3.81	Cell structure
CRY1	−3.81	Circadian clock
ACTA1	−3.78	Cell movement
CRYAB	−3.66	Apoptosis and oxidative stress
CRYBA1	−3.63	Lens function

## Discussion

The purpose of this study was to characterize the effects of spaceflight on the retinal vasculature and possible alterations in BRB integrity, and to identify spaceflight-induced proteomic significance and biomarkers in mouse ocular tissue. The data demonstrate that spaceflight induces apoptosis in the retinal vascular endothelial cells and photoreceptors (Fig. 2), as well as evokes alterations in vascular levels of AQP-4 (Fig. 3), PECAM-1 (Fig. 4) and ZO-1 (Fig. 5), proteins related to BRB integrity. These changes in BRB characteristics suggest decrements in barrier function in the eye. Protein expression profiles and pathway analysis also provide evidence that spaceflight induces changes in cellular organization, cell cycle, mitochondrial function, circadian clock and oxidative stress in the retina. Collectively, these observations are consistent with and extend previous findings in rodents exposed to a weightless environment^4,5^ and suggest that spaceflight involves a complex combination of stressors that leads to alterations and impairment of ocular structure and function.

Little is known about the impact of spaceflight on the BRB, but in disease states such as diabetes, where the BRB is compromised and microvascular permeability is elevated, visual impairment similar to the visual disturbances experienced by astronauts often occurs^9,10^. At least one recent study has shown that a compromise in the integrity of the vascular BRB causes vision-threatening vascular dysfunction^11^. Damage to microvascular endothelial cells is one of the major factors thought to underlie the increased permeability of the BRB in diabetic patients^9,10^. Radiation, another environmental stressor inherent to spaceflight, also induces disruption of endothelial barrier integrity^12,13^. In the present study, we found the spaceflight induced significant changes in the expression of several proteins related to BRB integrity and function, including APQ-4, PECAM-1 and ZO-1. The water channel protein AQP-4 has a crucial role in maintaining the integrity of BRB by forming a functional complex at astrocytic endfeet membranes^14^. The upregulation of AQP-4 in flight retinal GCL and INL indicate the potential for barrier integrity disruption and edema formation. PECAM-1 is highly expressed at endothelial cell-cell junctions, where it functions as an adhesive stress-response protein to maintain endothelial cell junctional integrity^15^. It is important for leukocyte-endothelium interactions, including the migration of leukocytes across the endothelium during inflammation^16^. PECAM-1 has also been shown to play a critical role in the pathogenesis of vascular degeneration^17^. Increased expression of adhesion molecules in endothelial cells, including PECAM-1, has also been associated with BRB disruption after retinal vascular injury^18^. The higher PECAM-1 expression in retina following spaceflight may consequently facilitate inflammatory cells to cross the BRB due to prolonged changes in the BRB permeability^19^. The integrity of the BRB is also dependent on TJs, and possibly adherent junctions, among endothelial cells^20^. Many studies have shown that vascular permeability is altered by decreasing the levels of tight junction proteins, including ZO-1^21^. Indeed, the degradation of TJ-associated proteins in retinal vessels following injury has been shown to lead to retinal vascular disorganization and BRB breakdown^14^. We found a tendency for a lower expression of TJ-associated protein ZO-1, which is mostly expressed in endothelial and epithelial cells^22^. The greater apoptosis of retinal vascular endothelial cells coupled with alterations in retinal vascular AQP-4, PECAM-1 and ZO-1 levels in FLT mice collectively suggest that spaceflight increases the risk for BRB disruption, leading to possible ocular impairment and injury.

After returning from long-duration spaceflight missions, many astronauts have been diagnosed with ocular structural and visual changes^23^. Studies have shown that changes in the gravitational environment can have pronounced transient and sustained effects on the eye^24^. Microgravity-induced vision changes may result from the interaction of multiple physiological factors, including changes in IOP, fluid shifts, ocular geometry and cardiovascular alterations^25–27^. Similarly, increases in IOP have been documented in ground-based models of spaceflight, such as head-down tilt^28^.

As part of this study, we assessed IOP before and after spaceflight in fully conscious mice. Given the current evidence that BRB integrity is compromised by spaceflight (Figs 2–5), it was surprising to find a significant decrease in post-flight IOP in one eye of the FLT mice (Fig. 1) rather than an increase. There are several possible explanations for this apparent paradox. The first is whether the lower IOP in the left eye of FLT mice was biologically significant. The pre- and post-flight difference in the mean IOP of the left eye in the FLT mice was slightly less than 1 mmHg (Fig. 1). Likewise, the pre- and post-flight difference in the mean IOP of the left eye in the GC mice was also slightly less than 1 mmHg, although this difference was not statistically significant (Fig. 1). This suggests that a ± 1 mmHg margin may be within the biological variability of the measure in mice. This, along with the lack of difference in the pre- and post-flight IOP of the right eye in the FLT mice, indicates that spaceflight had little to no significant biological effect on IOP measured approximately 1 day after returning to Earth. In this instance, it is also possible that BRB disruption and increases vascular permeability may contribute to the pathology of the visual disturbances, like those experienced by astronauts, without increases of IOP. Second, it is possible that spaceflight-induced increases in IOP are transient and no longer evident 28 hours after splash down. Confirmation of this possibility requires further measures of IOP during spaceflight. A third possible explanation for the apparent paradox of BRB disruption with no increase in IOP is that the magnitude of BRB alterations found with 35 days of spaceflight were not sufficient to elicit elevations in IOP. And finally, the disruption of BRB integrity coupled with the hemodynamic response to spaceflight in mice may have been insufficient to evoke an increase in IOP. For example, spaceflight in humans is thought to induce a cephalad fluid shift that can affect the flow and fluid pressures within the extracranial, intracranial and intraocular circulations, as well as within the cerebrospinal and aqueous humor fluid systems^29^. Similar cephalad fluid shifts have also been described in the hindlimb unloaded rat^30–32^, a ground-based rodent model to simulate a weightless environment. However, mice have a negligible hydrostatic pressure gradient on Earth owing to their small body size and do not show the same shifts in fluid volume during hindlimb unloading as that found in rats^33^. Additionally, during 30 days of spaceflight on the Bion-M1 satellite, mean arterial blood pressure in the aortic arch of mice is not altered^34^, and arteries in the head and hindlimbs of mice flown on the Bion-M1 and Space Shuttle do not show any structural remodeling to indicate chronic changes in fluid pressure associated with a cephalad fluid shift^26,27,35^. Thus, an impairment of BRB integrity in the eyes of mice during spaceflight may not result in an increase in IOP due to the lack of a cephalad fluid shift, as might be reasonably expected to occur in astronauts. This observation highlights one of the major limitations of utilizing mice to study the effects of spaceflight on bodily function instead of the rat.

Proteomic analysis of the retina, optic disc and optic nerve also suggests that spaceflight has adverse effects on the eye. For example, proteomics analysis revealed that SCY 3-like protein (SCYL3), also known as the protein-associating with the C-terminal domain of Ezrin (PACE-1), was the most down-regulated protein in the flight mice compared to controls. SCYL3 is proposed to regulate membrane protein or lipid trafficking at the Golgi and plasma membrane^36^. More recent studies have shown that SCYL3 plays an important role in regulating neuronal function and survival in mice and humans^37,38^. Pleiotrophin (PTN), also known as heparin-binding brain mitogen (HBBM), is one of the most up-regulated proteins in our proteomic analysis. This protein is found predominantly in the central nervous system (CNS), where it likely plays a role in tumor development including differentiation, angiogenesis, invasion, apoptosis and metastasis^39^.

**Figure 10:**
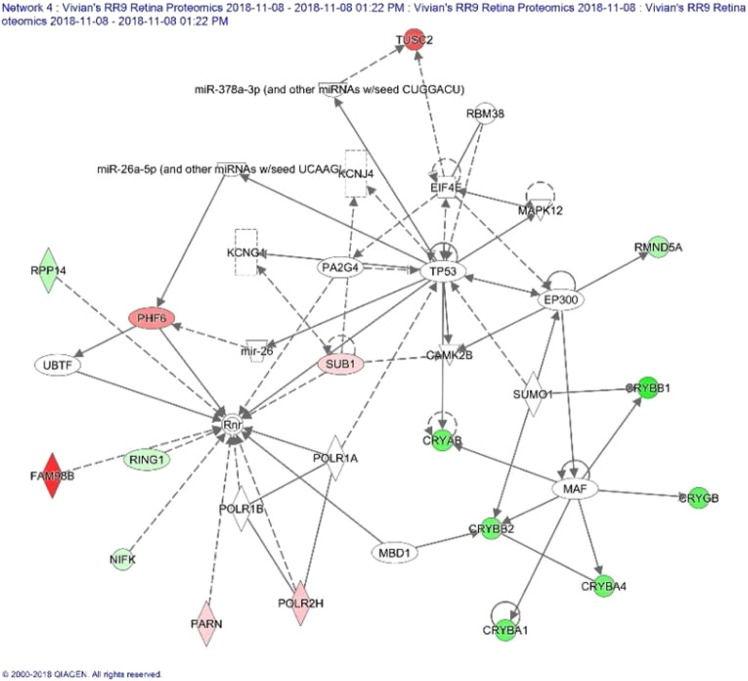
Impact of flight condition on expression of crystallins in the network of proteins associated with ophthalmic disease as defined by Ingenuity Pathway Analysis (IPA, https://www.qiagenbioinformatics.com/products/ingenuitypathway-analysis). Red = up-regulated (p < 0.1). Grey = un-changed. Green = down-regulated (p < 0.1). This figure is generated with IPA software. —Direct interaction. ╌╌╌╌ Indirection interaction. → Activation, expression, location, modification, molecular cleavage, phosphorylation, protein-DNA interactions, protein-RNA interactions, regulation of binding, transcription. ◊ Enzyme.  Ion Channel. A more detailed description of the network illustration (squares, circles, etc.) can be found at the Ingenuity Systems website. http://qiagen.force.com/KnowledgeBase/articles/Basic_Technical_Q_A/Legend.

Many proteins that are associated with ophthalmic disease and injuries were significantly altered by spaceflight, including proteins in the crystallins family (CRYAA, CRYAB, CRYGA and CRYBA1). These proteins are important in contributing to retinal ganglion cell survival^40^. Reduced expression of these proteins may indicate increased risk for developing visual diseases. Figure 10 illustrates regulation and interaction of network of proteins responsible for ophthalmic disease and abnormalities as defined by IPA in response to spaceflight.

Circadian rhythms recur regularly over an approximately 24-hour cycle affecting many aspects of biological processes in living beings ranging from tiny microbes to higher order animals, including humans^41^. In this study, the expression of CRY1, also known as cryptochrome, an important clock protein, was significantly down regulated by spaceflight. A recent study showed that CRY encodes a critical circadian photoreceptor^42^. Findings indicate that CRY regulates the light-mediated entrainment and the daily rhythms of locomotion^43,44^.

The overall results obtained in control mice showed there were no significant differences among GC, FV and GV control groups, suggesting that the time difference between tissue collection for the flight and ground cohorts did not have a significant impact on the measured endpoints. Therefore, we focused on comparisons between FLT and GC groups for most IHC assessments and the proteomics analysis, since these two groups shared the same flight hardware for housing and consumed the same food specially made for spaceflight. Additionally, the GC mice were exposed to the same environmental parameters as the FLT animals, including temperature, humidity and CO_2_ levels that were matched to the spaceflight condition.

Because of logistical limitations of spaceflight experiment, mouse IOP was measured and euthanasia was performed 28–38 hours after splashdown. We understand this to be a limitation of the study for all measured parameters in evaluating spaceflight-induced changes. However, we speculate that the structural and proteomic changes observed would be minimally affected by the time delay because protein expression is relatively stable and often requires days to weeks to be altered in response to environmental changes. In contrast, fluid pressures such as IOP can change in a matter of seconds to minutes.

In a previous investigation we reported the impact of 35 days of spaceflight on the mouse retina in animals which were housed in the mouse Habitat Cage Unit of the Japan Aerospace Exploration Agency (JAXA) “Kibo” facility on the ISS^5^. Results from the current paper are in agree with this previous study that spaceflight induces significant apoptosis in the retina and retinal vascular endothelial cells and some proteins and pathways associated with cell death, inflammation and metabolic stress were significantly altered following spaceflight Additionally, the current finding examine the molecular and cellular mechanisms of spaceflight-induced changes and provide strong evidence that spaceflight impacts BRB integrity. However, the proteomics analysis showed limited overlap of significant differentiating proteins and pathways between these two flight studies. For example, in the prior study with JAXA mice there were alterations in the glycogen degradation, fatty acid β oxidation and DNA methylation and transcription pathways of the flight mice that were not identified in the current study. In contrast, changes in the actin cytoskeleton, tight junction, and epithelial adherens pathways were significantly altered with spaceflight in the present study that were not observed in the JAXA flight mice. Some contrasting proteomic results between the two flight studies may not be surprising since several experimental conditions were different. First, for the JAXA mouse study, the posterior half of the right eyecup containing the retinal layer, optic disc and a short segment of the optic nerve was used for proteomic analysis, whereas the eye sample evaluated in the current study only contained the retinal layer. And secondly, 9-week old C57BL/6 male mice were used for the JAXA flight study, and the flight animals and control animals were obtained from breeding colonies in the United States and Japan, respectively. It is possible, although unlikely, that some of the differences in the proteomic results between flight and control animals reflected differences in mice from the two different breeding colonies. The RR-9 mice were obtained from the same US breeding colony. Thus, we believe that differences in the effects of spaceflight on protein expression and pathways in the eyes of mice between the previous JAXA study^5^ and the current study primarily reflect differences in the composition of the eye samples tested. Such differences may be important in guiding our understanding of the regional effects of spaceflight in the eye.

In conclusion, the results of this study demonstrate that exposure to a spaceflight environment is associated with increased retinal endothelial cells and photoreceptor cell death. In addition, there were alterations in the retinal microvasculature BRB integrity. Proteomic analysis showed that many key proteins and pathways responsible for cell death, cell organization, circadian clock regulation, inflammation, and metabolic stress were significantly altered. Multiple factors could be responsible for the changes observed in the FLT mice, including 35 days of weightlessness, increased radiation exposure, and distress associated with live return through the Earth’s atmosphere, splashdown in the Pacific Ocean, transport to the LLU laboratory, and approximately 38 hours of re-exposure to Earth’s gravity. The data further indicate that astronauts may be at risk for retinal damage and late degeneration. Such findings may also help facilitate the development and testing of more effective countermeasures for human spaceflight and planetary exploration.

## Material and Methods

### Flight and control conditions

On August 14, 2017, SpaceX successfully launched the 12th Commercial Resupply Service (CRS-12) payload at the Kennedy Space Center (KSC) which included 10-week-old male C57BL/6 mice (n = 20) (Jackson laboratories, Inc. Bar harbor, ME) for NASA’s ninth Rodent Research experiment (RR-9). The mice lived in NASA’s Rodent Habitats (RH) aboard ISS for 35 days before returning to Earth via SpaceX’s Dragon capsule. All mice were maintained at an ambient temperature of 26–28 °C with a 12-h light/dark cycle during the flight. GC mice were placed into the same housing hardware used in flight, and environmental parameters such as temperature and carbon dioxide (CO_2_) levels were kept equivalent to that of FLT mice based on 48 h delayed telemetry data. GC mice were fed the same reformulated rodent food bar diet approved by NASA as the space flown mice. Consumption of food and water was monitored daily throughout this study.

Animal experiments were approved by the Institutional Animal Care and Use Committee (IACUC) of Loma Linda University (LLU) and The National Aeronautics and Space Administration (NASA) and activities involving vertebrate animals are carried out in strict accordance with the recommendations in the Guide for the Care and Use of Laboratory Animals of the National Institutes of Health (NIH).

Due to Hurricane Irma’s impact on KSC in September, 2017, experiments with both GC and GV control groups were cancelled and later rescheduled for May, 2018, using mice of the same strain, sex, age and animals were from the same holding room from the vendor that were used for the flight experiments. This resulted in a large time gap between tissue collection for the flight and KSC ground control groups. Consequently, the FV cohort-matched control group was added to the study to help control for possible differences associated with this delay. Tissue collection with the FV group were conducted several days after studies with the FLT animals were concluded. Table 2 details the groups, housing conditions and mouse bodyweights for this study.

**Table 2:** Experiment groups, housing condition and body weights.

Group	Gravity	Number of mice	ACF	Housing	Food	Euthanasia Date	Body Weight at Mission (g)	Body Weight at Euthanasia (g)
Flight (FLT)	µg	20	ISS	RH	Bar	9/18/2017	25.6 +/− 0.5	26.6 +/− 0.5
Flight Vivarium Control (FV)	1 g	20	Vendor	VIV	Chow	9/22/2017	26.7 +/− 0.5	28.5 +/− 0.5
Ground Control (GC)	1 g	20	KSC	RH	Bar	6/18/2018	27.9 +/− 0.5	31.8 +/− 0.6
Ground Vivarium Control (GV)	1 g	20	Vendor	VIV	Chow	6/24/2018	26.1 +/− 0.6	30.0 +/− 0.3

ISS: International Space Station, KSC: Kennedy Space Center, ACF: animal care facility, RH: rodent habitat cage, VIV: vivarium cage, Bar: NASA Rodent Nutrient Food Bars, Chow: standard chow.

### Post-flight evaluation of the mice

Upon return to the Earth, mice were transported to LLU within 28 hours of splashdown. At LLU, animals were removed from the animal enclosure hardware and assessed for survival and health. The inspecting personnel reported that all the mice survived the 35-day space mission and were in good condition, i.e. no obvious deficiencies/abnormalities. Within 38 +/− 4 hours of splashdown, mice were euthanized.

### IOP measurement

Before launch and again before the post-flight euthanization, a handheld tonometer (TonoLab-ICARE, Raleigh, NC) was used to measure IOP in conscious animals. This allowed IOP to be measured accurately, rapidly and without a local anesthetic. Measurements of IOP using the rebound tonometer appeared to be well tolerated by the mice. The animals exhibited no signs of irritation or discomfort during the procedure. Five-6 sequential measurements were made to obtain an average of the counts as a single IOP readout for each mouse^45^. This process was repeated three times for each eye.

### Eye and retina preparation after spaceflight

The mice were rapidly euthanized in 100% CO_2_ and eyes were collected within 38 +/− 4 hours of splashdown (n = 20/group). The left eyes were fixed in 4% paraformaldehyde in phosphate buffered saline (PBS) for immunohistochemistry (IHC) assays. The right eyes from 2 experimental groups: GC and FLT were dissected to obtain the retina, and snap frozen in liquid nitrogen and stored at −80 °C for proteomics analysis.

### TUNEL assay

The ocular sections were evaluated using the TUNEL assay according to standard procedures. Data were obtained for 6 ocular samples of each group. Briefly, six μm paraffin embedded sections were processed using DeadEnd™ Fluorometric TUNEL system kit (Catalog No. G3250, Promega Corp., Madison, WI, USA) The same sections were then incubated with DyLight 594 Lycopersicon esculantum-Lectin (Vector Laboratories, Burlingame, CA, USA) to stain the endothelium. Nuclei were counterstained with diamidino-2-phenylindole (DAPI, blue, Life Technologies, Eugene, Oregon). Six to 10 field images were examined using a BZ-X710 All-in-One inverted fluorescence microscope with structural illumination (Keyence Corp., Elmwood Park, NJ) at 20X magnification spanning the entire retina per section.

### Immunohistochemistry for AQP-4 and vascular double-labeling

Six μm sections were incubated overnight (18–21 hours) at 4 °C with primary antibodies polyclonal rabbit anti-AQP-4 (1:500, Santa Cruz Biotechnology, Inc. Dallas, TX) and monoclonal mouse anti-glial fibrillary acidic protein (GFAP) clone GA5 (1:1000, Millipore, Burlington, MA) in 0.25% BSA, 0.25% Triton X-100 in PBS. Sections were washed 3 times in PBS and further treated with secondary antibodies Alexa Fluor 488 goat anti-rabbit IgG and Alexa Fluor 568 Goat anti-mouse IgG (1:1000, Life Technologies). Cell nuclei were counterstained with DAPI (1 μg/ml, Invitrogen) and sections were mounted and coverslipped with Vectashield Hard-Set Mounting Medium (Vector Laboratories, Burlingame, CA). Six to 10 field images were captured with a BZ-X700 inverted fluorescence microscope (Keyence Corp.) at 20X magnification spanning the entire retina sections.

### Immunostaining assays for PECAM-1 and ZO-1

Immunofluorescence staining related to BRB integrity on ocular sections was performed using biomarkers against PECAM-1 and ZO-1. Six µm sections were deparaffinized in Histoclear, rehydrated and washed in PBS for 20 minutes. For enzymatic antigen retrieval, sections were incubated in 1 mg/ml Actinase E (Sigma-Aldrich, St. Louis, MO) in PBS for 20 minutes at 37 °C, rinsed in running tap water for 3 minutes and washed in PBS for 5 minutes. Sections were then blocked in 1% BSA/PBS for 1 hour at room temperature. Vasculature was labeled with DyLight® 488 Lycopersicon Esculentum (Tomato) Lectin (1:100, Vector Laboratories) for 30 minutes at room temperature followed by 10 minutes wash in PBS. Sections were then incubated overnight at 4 °C with primary rabbit antibodies against ZO-1 (1:50) (Thermo Fisher Scientific, Hampton, NH) or CD31/PECAM-1 (1:100) (Novus Biologicals, Centennial, CO) in antibody dilution buffer (0.25% BSA, 0.25% Triton X-100 in PBS). After 3 washes in PBS, sections were incubated for 1.5 hours with secondary antibody goat anti-rabbit IgG Alexa Fluor® 568 (1:1000 in antibody dilution buffer; Life Technologies) followed by PBS washes. The cell nuclei were counterstained with DAPI solution (1 µg/ml in PBS; Life Technologies) washed in PBS and coverslipped with Vectashield® HardSet mounting medium (Vector Laboratories). Six to 10 field images were captured with a BZ-X700 inverted fluorescence microscope (Keyence Corp.) at 20X magnification spanning the entire retina sections.

### Quantification of Immunostaining

For quantitative analysis, numbers of TUNEL positive cells and TUNEL positive endothelial cells were counted separately. The total number of TUNEL-positive nuclear /ganglion cells or endothelial cells in the retina were counted in 5 sections from each eye. The surface of each section was measured on digital microphotographs using ImageJ v1.4 software (available as freeware from National Institutes of Health, Bethesda, MD, USA; http://rsbweb.nih.gov/ij/), and the density profiles were expressed as mean number of apoptotic cells/mm^2^. The mean of the density profile measurements across 5 retina sections per eye was used as a single experimental value.

To determine AQP4, ZO-1 and PECAM immunoreactivity, fluorescence intensity was measured on 6 to 10 randomly selected fields on each section and calculated using ImageJ software. Fluorescence intensities for positive cells (red channel for ZO-1 and PECAM, and green channel for AQP-4) from the areas of interest were measured and data were extracted and averaged across 5 retina sections per eye within the group.

### Proteomics analysis

A total of 12 frozen retinas from 2 experimental groups (GC and FLT, n = 6 mice per group) were shipped to the University of Arkansas for Medical Sciences for proteomics analysis.

Protein lysates were prepared and 20 μg of protein lysate were resolved in a 4–20% Tris-Glycine SDS-PAGE (Bio-Rad Laboratories, Hercules, CA). The gel was cut into 12 thick gel slices (2 mm thick) for each sample and an in-gel trypsin digestion was performed as previously described in (Mao *et al*. 2018). Briefly, gel slices were destained in 50% methanol (Thermo Fisher Scientific), ammonium bicarbonate (Sigma-Aldrich, St. Louis, MO) at 100 mM. Once destained, the proteins were reduced in 10 mM Tris [2-carboxyethyl] phosphine (Pierce, Dallas, TX) and alkylated in 50 mM iodoacetamide (Sigma-Aldrich). Gel slices were prepared for trypsin digestion by dehydrating in acetonitrile (Thermo Fisher Scientific). An overnight trypsin digestion was performed by the addition of 100 ng porcine sequencing grade modified trypsin (Promega) in 100 mM ammonium bicarbonate (Sigma-Aldrich) and incubation at 37 °C for 12–16 hours. Peptide products were then acidified in 0.1% formic acid (Pierce Corp.) to stop the trypsin reaction. Tryptic peptides were separated by reverse phase Jupiter Proteo resin (Phenomenex, Torrance, CA) on a 200 × 0.075 mm column using a nanoAcquity UPLC system (Waters). Peptides were eluted using a 30 minute gradient from 97:3 to 65:35 buffer A: B ratio. [Buffer A = 0.1% formic acid, 0.5% acetonitrile; buffer B = 0.1% formic acid, 99.9% acetonitrile.] The peptide eluents were ionized by electrospray (2.15 kV), MS1 peptides were detected and subjected to MS/MS analysis using higher-energy collisional dissociation (HCD) on an Orbitrap Fusion Tribrid mass spectrometer (Thermo Corp.) in top-speed data-dependent mode. MS data were acquired using the FTMS analyzer in profile mode at a resolution of 240,000 over a range of 375 to 1500 m/z. MS/MS data were acquired using the ion trap analyzer in centroid mode and normal mass range with precursor mass-dependent normalized collision energy between 28.0 and 31.0.

### Bioinformatic analysis of proteomics data

Proteins were identified and quantified using MaxQuant (version 1.6.0.16, Max Planck Institute). MS/MS spectra were submitted to MaxQuant for database searching using the UniProtKB database (2018-06 release; restricted to Mus musculus; 81,557 entries). MS1 iBAQ protein intensities were obtained by performing a MS1 reporter type database search with trypsin digestion including up to three missed cleavages. Modifications included both fixed modifications (carbamidomethyl of cysteine) and variable modifications (oxidation on methionine and acetyl on N-terminus). The first search was set to 5 ppm precursor ion tolerance for a search against a contaminants database in order to identify commonly identified contaminants, such as keratin proteins. The main search against the mouse database was set to 3 ppm. Label-free quantitation with iBAQ with a minimum ratio of 1 was selected to obtain the MS1 intensity values. Peptide and protein identifications were accepted using the 1.0% false discovery rate identification threshold^46^.

The MaxQuant iBAQ values for each sample were median normalized so the medians were equal to the sample with the maximum median (sample FLT 5). Median normalized iBAQ intensities were then imported into Perseus (version 1.6.1.3, Max Planck Institute). Perseus was used to log2 transform the data and impute the missing values using a normal distribution with a width of 0.3 and a downshift of 2 standard deviations. Principal Component Analysis was performed on the normalized data set and a batch effect was detected among replicates due to sample processing (data not shown). The batch effect was corrected using the R function ComBat from the SVA Bioconductor package^7^. Non-parametric Mann-Whitney u tests were used to compare the ground GC and FLT sample groups. The p-values were corrected by Benjamini-Hochberg to account for multiple testing. Fold changes were also calculated. Proteins that were significant by the FDR-adjusted p-value < 0.05 and that had a fold change greater than 2 were considered to be differentially expressed. The heat maps were generated using the Bioconductor R package ComplexHeatmap with the Euclidean distance metric and the clustering method set to complete^47^. Volcano plots were generated using R base function plot (R version 3.4.4). These significant proteins were then analyzed using IPA (QIAGEN Inc., Frederick, MD).

### Statistical analysis

The results obtained from IOP measurements, TUNEL assay and IHC were analyzed by one-way analysis of variance (ANOVA) and Tukey’s HSD (honestly significant difference) test for multiple pair-wise comparisons (Sigma Plot for Windows, version 13.0; Systat Software, Inc., Point Richmond, CA). Means and standard error of means (SEM) are reported. The statistical analysis of proteomics results is described above.

## Supplementary information

Supplemental Table 1

Supplementary Table 2
